# Informatics methodology for the rapid, validated development of clinical research forms in CARRAnet: incorporating user-centered design into the Delphi technique

**DOI:** 10.1186/1546-0096-10-S1-A16

**Published:** 2012-07-13

**Authors:** CJ Inman, Marc Natter, Robert W Warren

**Affiliations:** 1 Children's Hospital of Boston, Boston, MA, USA; 2 Stanford, CA, USA; 3 Texas Children's Hospital, Houston, TX, USA; 4 University of Utah, Salt Lake City, UT, USA

## Purpose

CARRAnet is a multicenter and multiple disease national registry in pediatric rheumatology with up to sixty sites participating. The CARRAnet registry is designed as a framework from which to drive clinical research and creation of evidence based treatment protocols. Success of the project relies on the continued participation of the clinical sites. User centered design is an informatics technique for creating an effective and efficient system that creates positive attitudes among the end users^1^. We describe a methodological approach toward incorporating user centered design into the traditional Delphi process for data-element development as an approach for testing and validation of case report forms (CRF) for clinical registries.

## Methods

Common and disease specific data elements sets were created using a modified Delphi Technique by expert panels of pediatric rheumatologists^2^. Site Principal Investigators and study coordinators were contacted via email to participate in User Testing. Each user was sent a data element set, encompassing an initial and follow-up visit form along with a User Survey. A semi-structured interview was conducted over the phone by a researcher (CJI), qualitative information about missing, inaccurate or confusing data elements were recorded. The results of the user testing were recorded in a synopsis allowing anonymity to participants. The synopsis was a standardized summary of responses, in 3 domains and on a graded scale: who was providing feedback (attending, fellow or study coordinator), strength of opinion, and degree of agreement. This was presented to an executive panel and revisions to the data elements and instructions were made at their discretion. The accepted set of data elements and instructions were used for the registry CRFs.

## Results

A common and 7 disease specific data element sets each with an initial and follow up visit form were created. The common and 5 disease specific data element sets underwent user testing. There were a total of 44 participants including 27 physicians.

**Figure 1:**
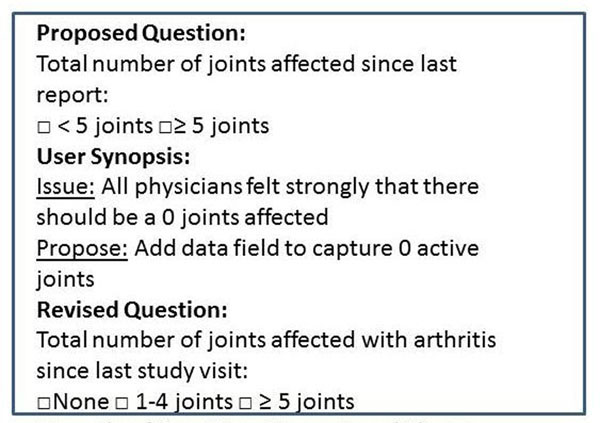
Example of question, synopsis and change

## Conclusion

We describe a methodology of incorporating user centered design into the Delphi Technique for creation of CRFs for the CARRAnet clinical registry. We propose that User Centered Design will encourage participation and provide more accurate data collection. Future research involves in-depth analyses of the content of the accepted versus rejected data elements based on user testing.

## Disclosure

C.J. Inman: None; Marc Natter: None; Robert W. Warren: None; CARRA Investigators: None.

